# Oxidative stress caused by a low concentration of hydrogen peroxide induces senescence-like changes in mouse gingival fibroblasts

**DOI:** 10.3892/ijmm.2012.1102

**Published:** 2012-08-20

**Authors:** TAMOTSU KIYOSHIMA, NORIO ENOKI, IEYOSHI KOBAYASHI, TAKAKO SAKAI, KENGO NAGATA, HIROKO WADA, HIROAKI FUJIWARA, YUKIKO OOKUMA, HIDETAKA SAKAI

**Affiliations:** 1 Laboratory of Oral Pathology and Medicine, Faculty of Dental Science, Kyushu University, Fukuoka 812-8582;; 2 Department of Morphological Biology, Pathology Section, Fukuoka Dental College, Sawara-ku, Fukuoka 814-0193;; 3 Departments of Fixed Prosthodontics and; 4 Pediatric Dentistry, Faculty of Dental Science, Kyushu University, Fukuoka 812-8582, Japan

**Keywords:** stress, gingival fibroblast, cell senescence

## Abstract

Periodontal tissue deteriorates under persistent oxidative stress induced by inflammatory reactions in the microflora of the oral cavity. This study aimed to evaluate the cellular properties of mouse gingival fibroblasts (MGFs) in the presence of oxidative stress. MGFs from 10-, 30- and 52-week-old mice were used to evaluate the changes in the cellular properties with aging. The study investigated the effects of oxidative stress on the cellular properties of MGFs from 10-week-old mice. The expression of p53, p21 and murine double minute 2 (Mdm2) in the MGFs in response to oxidative stress was also examined. By day 8, the number of MGFs increased in culture. However, the increase was markedly lower in MGFs derived from aged mice. Oxidative stress due to hydrogen peroxide (H_2_O_2_)-induced morphological changes characterized by a round shape with enlarged nuclei and expanded cytoplasm. The cell number of MGFs was decreased subsequent to treatment with 50 μM or a higher concentration of H_2_O_2_. MGFs treated with H_2_O_2_ at 20 μM showed a similar cell growth curve as the one seen in 52-week-old mice. Phosphorylated p53 protein was increased in MGFs subsequent to treatment with 20 μM H_2_O_2_, along with an upregulated transcription of p21 and Mdm2 mRNAs. These results suggest that treatment with a lower concentration of H_2_O_2_ in MGFs induces cell cycle arrest, resulting in stress-induced premature senescence, possibly correlated with the development of periodontal diseases.

## Introduction

Aging is a physiological phenomenon, which commonly occurs in various organs and tissues (1). Age-dependent morphological and cell kinetic changes in the organs and tissues are associated with the development of various diseases in the elderly. A decline in the function of organs and tissues is a normal phenomenon associated with aging, and is also considered to reduce the quality of life (1).

Several reports are available regarding the relationship between aging and periodontitis (2–5). The aging of the periodontal tissue is involved in the development of periodontitis in elderly individuals (6).

The age-dependent morphological and cell kinetic changes of the gingival tissue were delineated, using both the 5-bromo-2′-deoxyuridine (BrdU) incorporation and the terminal deoxynucleotidyl transferase-mediated deoxyuridine-5′-triphosphate (dUTP)-biotin nick end-labeling (TUNEL) methods (7,8). Those studies demonstrated that with aging there is a significant apoptosis-induced decrease in the cellular component of the subepithelial connective tissue of both the gingival and junctional epithelial layer. Furthermore, an age-dependent increase in the number of TUNEL-positive cells occurred only in the subepithelial connective tissue, although gingival tissue, buccal mucosa, tongue dorsal, ventral mucosae and skin have similar histological structures (9).

Oxidative stress is one of the most important causative factors for the induction of cell apoptosis (10,11). Incubation-induced subcytotoxic stress with potentially harmful molecules, such as hydrogen peroxide (H_2_O_2_) brought the cells into a state similar to senescence, termed stress-induced premature senescence (SIPS) (12–14). Therefore, in this study, the age-dependent changes in the cell number in cultured mouse gingival fibroblasts (MGFs), as well as the changes in the biological behavior subsequent to treatment with H_2_O_2_ in the MGFs were investigated.

## Materials and methods

### Animals

BALB/c mice were used to investigate the age-related changes in the cell number in MGFs in 10-, 15-, 30- and 52-week-old mice (n=3 per group). In addition, 10-week-old mice were used for the oxidative stress experiment (n=3 per group). The animals were bred in a strictly monitored air-conditioned clean room and were fed standard laboratory pellets and water *ad libitum*. The experiment was performed according to the guidelines of the Animal Center at the Kyushu University.

### Gingival fibroblast cultures

The animals were sacrificed using an excessive amount of ether. The gingival tissues were removed, immediately washed in phosphate-buffered saline (PBS) with gentamicin (10 μg/ml; Invitrogen, Carlsbad, CA, USA) and transferred into a culture dish. The gingival fibroblasts were grown in α-MEM, supplemented with 10% fetal bovine serum (FBS), 100 IU/ml penicillin and 100 μg/ml streptomycin in a humidified atmosphere with 5% CO_2_ at 37°C. The medium was changed every other day. The cells were grown to semi-confluence, harvested by trypsinization at 37°C for 5 min, then subcultivated with culture medium in a new dish. The experiments were performed using early-passage fibroblasts before the fourth passage.

### Cell growth assay

MGFs (1.0×10^4^ cells/well) were seeded onto 12-well plates in the α-MEM with serum. The cell numbers in three-wells of each group were counted chronologically at 2, 4, 6 and 8 days after cultivation. The results were expressed as the mean ± SD.

### Oxidative stress

At 48 h after seeding, MGFs were exposed to oxidative stress for 2 h. Various concentrations of H_2_O_2_ were diluted in α-MEM with 10% FBS. Subsequent to treatment with H_2_O_2_, the cultures were rinsed twice with PBS and incubated in α-MEM with 10% FBS. Some samples were stained with nuclear fast red to observe cellular and nuclear morphological changes in the MGFs after the oxidative stress.

### Senescence-associated β-galactosidase (SA-β-Gal) activity

MGFs were cultured for 14 days subsequent to treatment with H_2_O_2_. The SA-β-Gal-positive cell ratios were determined in three wells, using a senescence detection kit, according to the manufacturer’s instructions. To avoid any non-specific staining due to confluence, SA-β-Gal cytochemical staining was performed on non-confluent cells.

### Semi-quantitative reverse transcriptase polymerase chain reaction (RT-PCR)

A semi-quantitative RT-PCR analysis was carried out. Total-RNA extracted from cultured mouse gingival cells was isolated using the SV total-RNA isolation system, according to the manufacturer’s instructions. cDNA was generated from isolated total-RNA by reverse transcription (RT) with SuperScript III and subjected to PCR amplification with the specific primer sets (Table I). Glyceraldehyde-3-phosphate dehydrogenase (GAPDH) was used as the internal RNA control for the comparison of RNA levels in each sample. The PCR products were separated by electrophoresis on agarose gels and then stained with ethidium bromide.

### Immunoblotting for the p53 phosphorylation status

An immunoblot analysis for p53 was carried out using the proteins isolated from the cultured MGFs after the treatment with 20 μM H_2_O_2_ for 2 h. These cells were lysed in RIPA buffer (50 mM Tris pH 8.0, 150 mM NaCl, 1% Triton X-100, 1 mM EDTA pH 8.0, 0.1% SDS), supplemented with protease inhibitor cocktail (50 μM), lactacystin (20 μM) and PMSF. The protein samples were separated using SDS-PAGE, transferred to an Immun-Blot^®^ PVDF Membrane, and immunoblotted with anti-p53 antibody (sc-6243; Santa Cruz Biotechnology, Inc., Santa Cruz, CA, USA) and anti-phospho-p53 antibody (S15; R&D Systems, Inc., Minneapolis, MN, USA). The membrane was incubated with suitable secondary antibody conjugated with horseradish peroxidase. Immunoreactive proteins were visualized using an ECL detection system. Any emitted light was detected using a cooled CCD-camera (LAS-1000; Fujifilm, Tokyo, Japan).

### Statistical analysis

The experiments were repeated three times for the independent MGF samples. A statistical analysis was performed combining one-way ANOVA with the Tukey-Kramer comparison test or Student’s t-test. P-values <0.05 or 0.01 were considered to indicate statistically significant differences.

## Results

### Age-dependent change in the cell number in MGFs

**Figure 1.**
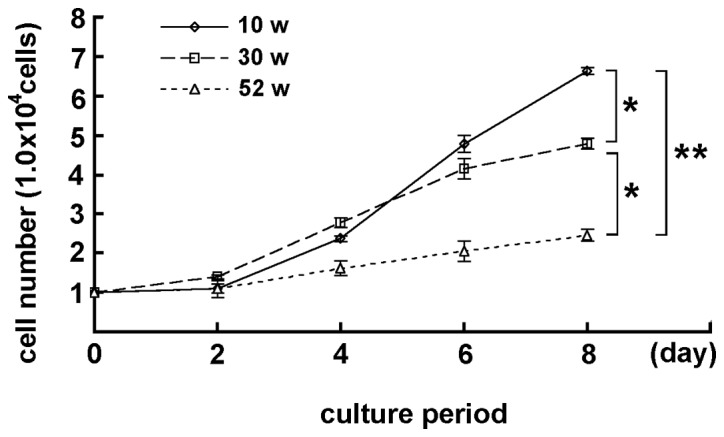
Chronological change of the number of MGFs isolated from 10-, 30- and 52-week-old mice. The cell growth curve of MGFs at each culture day was different at each age. Significant differences in the cell number at day 8 of culturing were detected between 10- and 30-week-old, and 30- and 52-week-old mice (^*^P<0.05). A significant difference was also observed between 10- and 52-week-old mice (^**^P<0.01). The cell growth curves of the MGFs are different in the 10-, 30- and 52-week-old mice.

MGFs isolated from 10-, 30- and 52-week-old mice were cultured for 2, 4, 6 and 8 days. Although the MGFs showed cell proliferation while cultured, the number of MGFs was different on each culture day, depending on the age of the mouse, from which the sample was derived. Marked differences were detected in the cell number in 10- and 30-week-old, and 30- and 52-week-old mice (P<0.05) at day 8 of the culturing. A statistically significant difference was also observed between 10- and 52-week-old mice (P<0.01), indicating that the increase in the number of cultured MGFs was apparently lower in samples from older mice (Fig. 1). In the subsequent oxidative stress experiments MGFs derived from 10-week-old mice were used.

### Effects of oxidative stress on the morphological change in MGFs subsequent to H_2_O_2_ treatment

**Figure 2.**
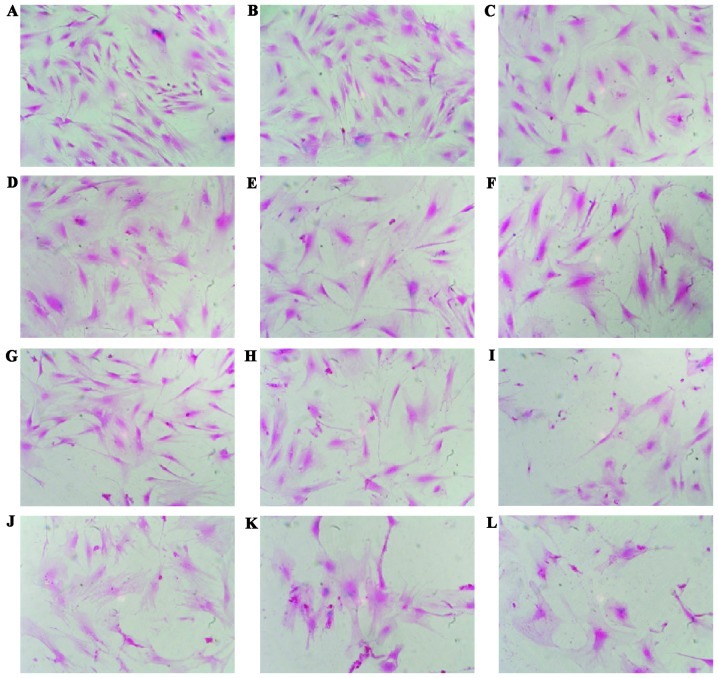
Morphological change in MGFs subsequent to treatment with H_2_O_2_ is shown. MGFs were (A) non-treated and (B–L) treated with various concentrations of H_2_O_2_. H_2_O_2_-treated cells showed a round shape with enlarged nuclei and expanded cytoplasm. A decrease in the cell number was evident in the H_2_O_2_-treated samples. (A) Non-treated, (B) 50 μM, (C) 100 μM, (D) 150 μM, (E) 200 μM, (F) 250 μM, (G) 300 μM, (H) 350 μM, (I) 400 μM, (J) 450 μM, (K) 500 μM and (L) 550 μM.

MGFs were treated with various concentrations of H_2_O_2_. The MGFs were observed under the microscope after 24 h. Non-treated MGFs showed a fibroblastic spindle shape. As seen in previously-reported SIPS-like changes, the H_2_O_2_-treated cells had a round shape with enlarged nucleus and expanded cytoplasm (Fig. 2) (13,14). The detachment of the cells from the culture dish also increased in an H_2_O_2_ concentration-dependent manner, indicating that cell death occurred in the MGFs.

### Effects of oxidative stress on the cell number of MGFs subsequent to H_2_O_2_ treatment

**Figure 3.**
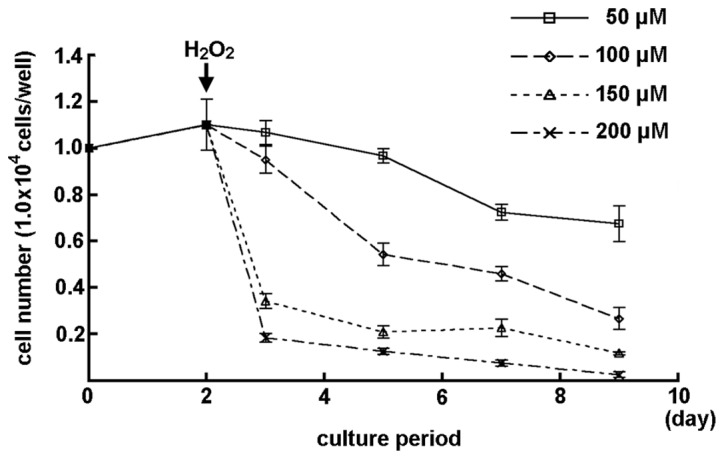
Effect of H_2_O_2_ treatment on the cell growth of MGFs. A slight decrease in the cell number was observed in the MGFs subsequent to treatment with 50 μM H_2_O_2_. Additionally, a marked decrease in the cell number in MGFs is detected subsequent to treatment with 100 μM or higher concentrations of H_2_O_2_.

The number of MGFs subsequent to treatment with various concentrations of H_2_O_2_ is shown in Fig. 3. A slight decrease in the cell number was observed in the MGFs treated with 50 μM H_2_O_2_. A marked decrease in the cell number of MGFs was detected subsequent to treatment with 100 μM or higher concentrations of H_2_O_2_ (Fig. 3).

### Effect of lower concentrations of H_2_O_2_ on the cell growth in MGFs

**Figure 4.**
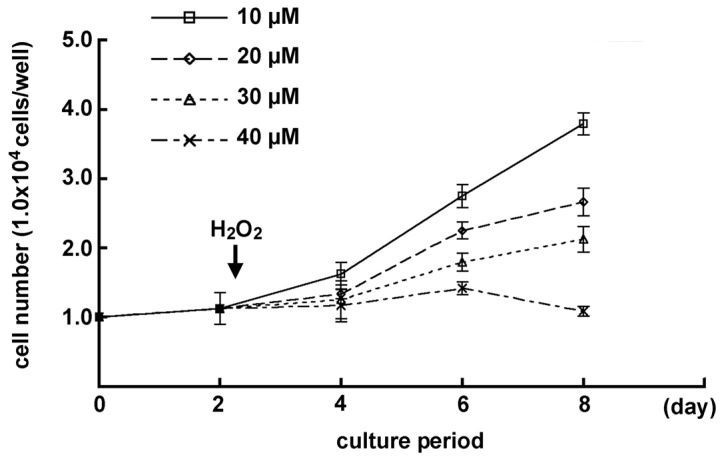
Effect of lower concentrations of H_2_O_2_ on the cell growth in MGFs. A chronological increase in the cell number was demonstrated in samples with 10, 20 and 30 μM H_2_O_2_. No significant change was seen in the cell growth in the sample after 40 μM H_2_O_2_-treatment.

The effects of low H_2_O_2_ concentrations on the MGFs were examined, since the cell number of MGFs decreased gradually when treated with 50 μM H_2_O_2_. A chronological increase in the cell number was demonstrated in samples with 10, 20 and 30 μM H_2_O_2_-treatment. MGFs treated with 20 μM H_2_O_2_ showed a chronological cell growth curve, similar to the one seen in the 52-week-old mice. By contrast, no significant change was detected in the cell number of the samples subsequent to treatment with 40 μM H_2_O_2_ by day 6 of culturing, while a decreased number of cells was observed at day 8. Statistically significant differences were demonstrated in each sample at day 8 of culturing (P<0.05) (Fig. 4).

### Effect of 20 μM concentration of H_2_O_2_ treatment on the cell growth in MGFs in a prolonged culturing

**Figure 5.**
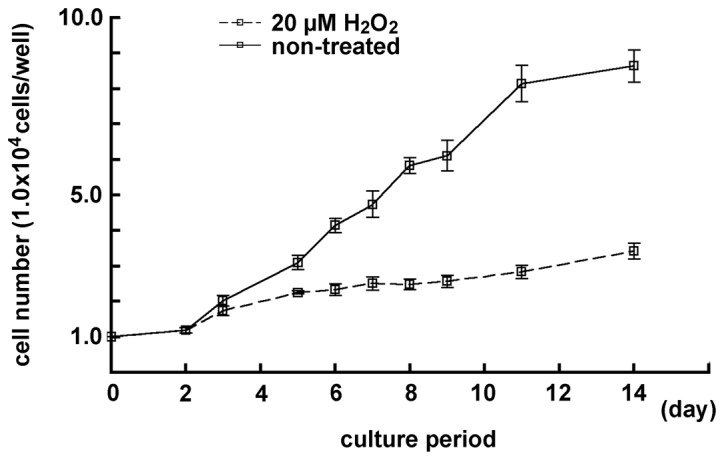
Effect of 20 μM concentration of H_2_O_2_ treatment on the cell growth in MGFs in a prolonged culturing. A slightly increased ratio in the cell number of MGFs treated with H_2_O_2_, was observed after 8 days of culturing. The growth curve is similar to the one observed in samples from 52-week-old mice.

The cell number in the samples treated with 20 μM H_2_O_2_ was examined and compared with the cell number in the non-treated samples, for a period prolonged by 14 days. After 8 days of culturing, the cell number in samples treated with a lower concentration of H_2_O_2_ decreased in the cultured MGFs, and a low cell number increase-ratio was observed. There was a notable difference between the non-treated and the treated samples (P<0.05) (Fig. 5). Furthermore, H_2_O_2_-treated cells had a round shape with enlarged nucleus and expanded cytoplasm, as observed in the senescent fibroblasts. Therefore, MGFs treated with 20 μM H_2_O_2_ seemed to mimic the age-dependent decrease of the proliferative activity in the 52-week-old mice.

### Detection of SA-β-Gal in the MGFs using a 20 μM H_2_O_2_ treatment

**Figure 6.**
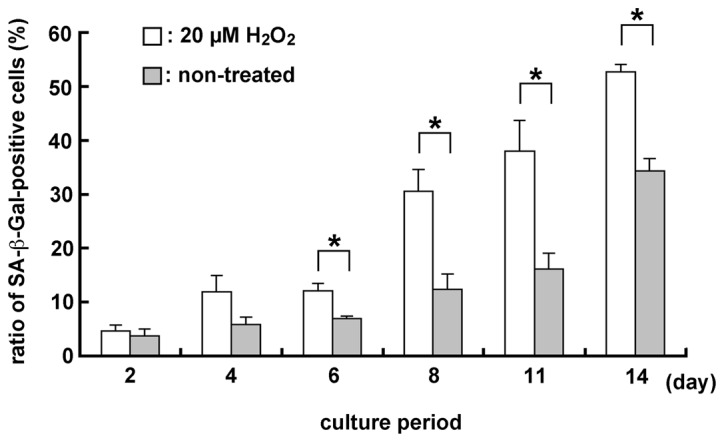
Detection of SA-β-Gal in the MGFs using 20 μM H2O_2_. Two days after the treatment, the ratio of SA-β-Gal-positive cells was larger in H_2_O_2_-treated samples than that in non-treated samples. Significant differences in the number of SA-β-Gal-positive cells were observed between the 20 μM H_2_O_2_-treated and non-treated samples cultured at 6, 8, 11 and 14 days of culture (^*^P<0.05).

The ratio of SA-β-Gal (a senescence marker)-positive cells was examined in H_2_O_2_-treated and non-treated samples, since oxidative stress after H_2_O_2_ treatment induced senescence-like morphological and functional changes. Two days after the treatment, the ratio of SA-β-Gal-positive cells was higher in H_2_O_2_-treated compared to non-treated samples (Fig. 6). Significant differences were noted in the number of SA-β-Gal-positive cells in 20 μM H_2_O_2_-treated and non-treated samples (P<0.05), subsequent to culture for 6, 8, 11 and 14 days.

### Expression of p53, p21 and Mdm2 mRNA, and the phosphorylation of p53 protein in the culture MGFs

**Figure 7.**
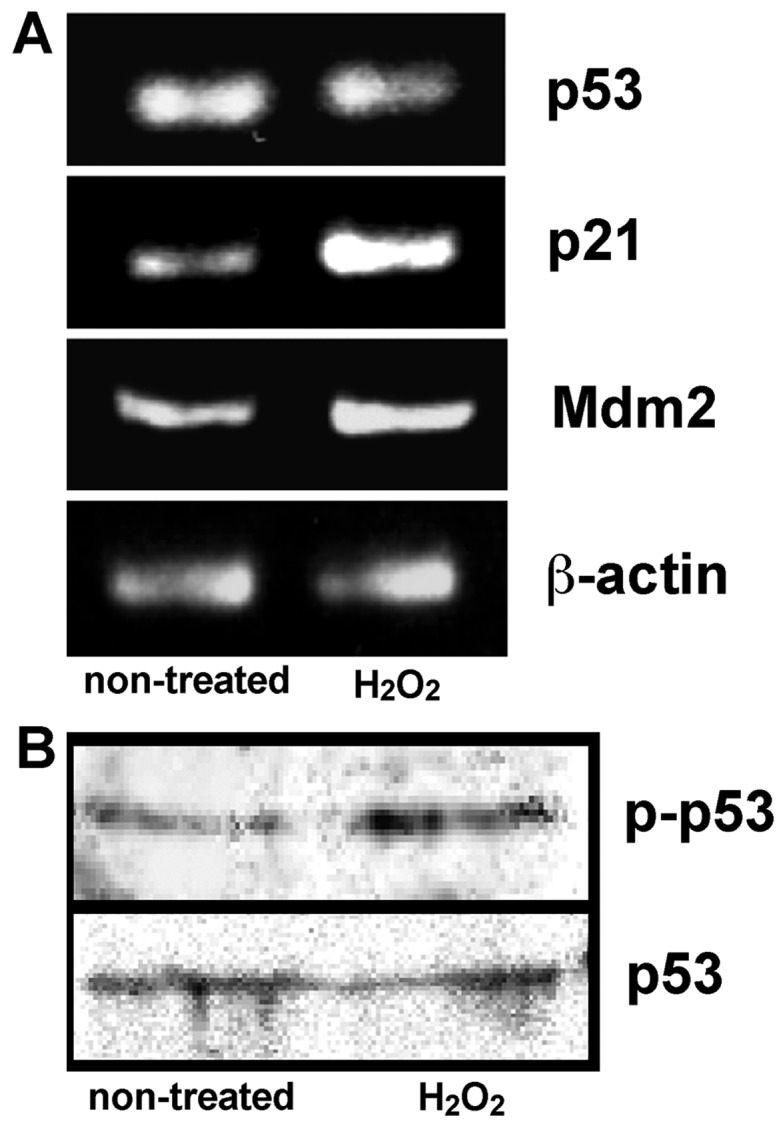
Expression of p53, p21 and Mdm2 mRNA, and the phosphorylation of p53 protein in the culture MGFs under oxidative stress. No significant change in either (A) mRNA or (B) protein expression of p53 was detected in the cultured MGFs treated with 20 μM H_2_O_2_. (B) An increased expression of phosphorylated p53 protein was observed in the sample treated with 20 μM H_2_O_2_. (A) An increased expression of p21 and Mdm2 mRNAs was also demonstrated in the same sample.

Oxidative stress induced senescence-like changes in the MGFs, and therefore the expression of p53 having the potential to initiate cell cycle arrest or cell death (15,16) was further examined. In addition, we examined the expression of p53 downstream genes, cyclin-dependent kinase inhibitor p21 and Mdm2 (17) was examined. The phosphorylated p53 protein was detected using an antibody specifically recognizing human, monkey and rat p53 phosphorylated at the serine-15 and the comparable phosphorylated site in mouse p53 (serine-18). By contrast, unphosphorylated p53 is not detected by the antibody. No significant change regarding the mRNA and protein expression of p53 was detected between the non-treated MGFs and MGFs treated with 20 μM H_2_O_2_, whereas an increased expression of phosphorylated p53 protein was observed in the samples treated with 20 μM H_2_O_2_. An increased expression of p21 mRNA was demonstrated in the same sample, thus indicating the induction of increased p21 expression by phosphorylated p53 protein. The Mdm2 mRNA was also increased in the cultured MGFs treated with 20 μM H_2_O_2_ (Fig. 7).

## Discussion

The present study demonstrated that oxidative stress generated by treatment with a lower concentration of H_2_O_2_ induced a decrease in the cell number in the cultured MGFs with an increased ratio of SA-β-Gal-positive cells, a marker of cellular senescence. Oxidative stress generated by externally added H_2_O_2_ induces SIPS in variety of cell types (13,14,18). This study demonstrated, for the first time, that SIPS in gingival fibroblasts was induced by a lower-concentration H_2_O_2_-treatment than previously reported. Previous reports have proven a low concentration of H_2_O_2_ treatment to induce a higher proliferative activity in human dorsal fibroblasts, rabbit lens epithelial cells, baby hamster kidney fibroblasts and embryonic Chinese hamster ovary fibroblasts (19–21). Therefore, it is reasonable to assume that the gingival fibroblast is more sensitive to H_2_O_2_ oxidative stress.

Oxidative stress is one of the most important causative factors for the induction of pathological states, including periodontitis (22–26). Periodontal tissue tends to deteriorate under persistent oxidative stress induced by inflammatory reactions in the microflora of the oral cavity (27). A previous report demonstrated that gingival fibroblast and dorsal fibroblast had different cellular properties, such as the expression of integrin and extracellular matrix receptors. Consequently, experiments using dorsal fibroblasts are not necessarily valid for oral tissues as well (28). These different cellular properties appear to be correlated with higher sensitivity to oxidative stress, thus resulting in the induction of SIPS.

The p53 gene expression was examined since gingival fibroblasts appeared to be more sensitive to oxidative stress. p53 activation is closely correlated with cell cycle arrest and apoptosis when cells deteriorate due to pathogenic stress. There was no change in the level of p53 mRNA and protein expression shown by the semi-quantitative RT-PCR analysis and western blotting, respectively. However, western blotting demonstrated an increase of phosphorylated p53 protein. Therefore, an increased p21 expression level, suppressing the CDK4- and CDK6-activation, is considered to be induced by the increase of phosphorylated p53 protein, resulting in a decrease of cell number in the MGFs treated with a lower concentration of H_2_O_2_. The Mdm2 expression was also activated at the transcription level. These results suggest that Mdm2 functions as a negative feedback regulator to maintain p53 at a low level under oxidative stress, since the major function of Mdm2 is to interact with p53 and thereby induce the ubiquitination and degradation of p53 (29).

In this study, H_2_O_2_-induced oxidative stress generated an increase in the phosphorylated p53 protein-level at serine-18 in the MGFs. The phosphorylation at this site in mouse p53 is comparable to that of human p53 at serine-15. Phosphorylation of human p53 at serine-15 has a significant role in its reduced interaction with the Mdm2, its negative regulator, and is involved in the impairment of the Mdm2 function by inhibiting p53-dependent transactivation (30). Therefore, the serine-18 of mouse p53 protein may be one of the critical phosphorylation sites that lead to cell cycle arrest and SIPS under oxidative stress. However, the H_2_O_2_-mediated protein kinases involved in p53 phosphorylation remain unknown. Nevertheless, these results do not exclude the possibility that phosphorylation at other sites on p53 may be associated with the occurrence of these phenomena.

Therefore, gingival fibroblasts are more sensitive to oxidative stress, resulting in cell cycle arrest due to an increase of phosphorylated p53 protein. This characteristic of the gingival fibroblasts may be associated with the marked age-dependent decrease of cellular components in the periodontal tissue described in previous studies (7–9), as well as with the development of periodontal diseases. In order to develop new preventive methods against various periodontal diseases, additional investigations regarding the molecular interactions within the gingival fibroblasts in association with oxidative stress are required.

## Figures and Tables

**Table I.** Primer sets used for semi-quantitative RT-PCR.

Target	Sequence
p53	F: 5′-GGA AAT TTG TAT CCC GAG TAT CTG-3′
	R: 5′-GTC TTC CAG TGT GAT GAT GGT AA-3′
p21	F: 5′-TGT CCA ATC CTG GTG ATG TC-3′
	R: 5′-TCT CTT GCA GAA GAC CAA TCT G-3′
Mdm2	F: 5′-CCA GGC CAA TGT GCA ATA C-3′
	R: 5′-GTG AGC AGG TCA GCT AGT TGA A-3′
GAPDH	F: 5′-ACC ACA GTC CAT GCC ATC AC-3′
	R: 5′-TCC ACC ACC CTG TTG CTG TA-3′

F, forward; R, reverse; Mdm2, murine double minute 2; GAPDH, glyceraldehyde-3-phosphate dehydrogenase.
